# Silencing RORγt in Human CD4^+^ T cells with CD30 aptamer-RORγt shRNA Chimera

**DOI:** 10.1038/s41598-019-46855-9

**Published:** 2019-07-17

**Authors:** Xiaofei Shi, Pingfang Song, Shao Tao, Xiaowei Zhang, Cong-Qiu Chu

**Affiliations:** 1 Division of Arthritis and Rheumatic Diseases, Oregon Health & Science University and VA Portland Health Care System, Portland, OR 97239 USA; 2 Department of Rheumatology and Immunology, The First Affiliated Hospital and College of Clinical Medicine, Henan University of Science and Technology, Luoyang, China

**Keywords:** Cytokines, Autoimmunity

## Abstract

Targeting specific T cell subtypes and intervening in their function are emerging a critical strategy for treatment of autoimmune diseases. Here we report that an RNA CD30 aptamer was utilized to deliver short hairpin RNA (shRNA) to CD30^+^ T cells to target retinoic acid receptor-related orphan receptor gamma t (RORγt), leading to impaired expression of RORγt and suppression of IL-17A and IL-17F. A DNA template consisting of CD30 aptamer and RORγt shRNA sequences was synthesized and was transcribed CD30 aptamer-RORγt shRNA chimera (CD30-AshR-RORγt). Insertion of 2′-F-dCTP and 2′-FdUTP was incorporated during CD30-AshR-RORγt transcription to increase its resistance to RNase. CD30-AshR-RORγt was specifically up-taken by CD30^+^ Karpas 299 cells, but not by Jurkat cells which lack CD30. It was also up-taken by activated, CD30 expressing human CD4^+^T cells, but not by resting CD4^+^ T cells. The RORγt shRNA moiety of CD30-AshR-RORγt chimera was cleaved and released by Dicers. Then, CD30-AshR-RORγt suppressed RORγt gene expression in Karpas 299 cells and activated human CD4^+^ T cells. Consistently, silence of Th17 cell differentiation and IL-17A and IL-17F synthesis with CD30-AshR-RORγt was demonstrated in activated human CD4^+^ T cells from healthy donors and RA patients. CD30-AshR-negative control chimera and prostate specific membrane antigen (PSMA)-AshR-RORγt had no significant impact on the expression of RORγt or IL-17A and IL-17F. These data present a novel strategy for shRNA delivery using CD30 RNA aptamers to down-regulate CD30^+^ Th17 cells and can be developed as a targeted therapy for treating Th17 cell mediated conditions.

## Introduction

T helper type 17 (Th17) cells are derived from naϊve CD4^+^ T cells which are induced by coordination of transforming growth factor (TGF)-β, interleukin (IL)-6, IL-21 and IL-23. Th17 cells express the master transcription factor, retinoic acid-related orphan receptor γ t (RORγt), and predominantly synthesize and release the signature cytokines, IL-17A and IL-17F^1–3^. It has been experimentally and clinically demonstrated that Th17 cells and their released cytokines exert a key role in the pathogenesis of autoimmune inflammatory diseases^3^. In human psoriasis, IL-17A blockade in established disease displays a striking therapeutic efficacy, indicating the critical contribution of IL-17A to the pathogenesis of psoriasis^4,5^. In comparison, the role of IL-17 in rheumatoid arthritis (RA) is complex. Data from animal models clearly indicate the critical role of IL-17A in the pathogenesis of experimental arthritis^6^. However, clinical trials with monoclonal antibodies blocking IL-17A in established RA patients showed no superior therapeutic effects than blocking tumor necrosis factor (TNF)^7,8^. These results and results from pre-clinical RA patients suggest that IL-17A may play a more critical role in the early stage of RA^9^. Alternatively, in RA, both IL-17A and IL-17F and other inflammatory cytokines released by Th17 cells are important in driving disease progression in RA. Therefore, blocking Th17 cell development may be more effective in treating RA. The master transcription factor, RORγt controls the final differentiation of Th17 cells and biosynthesis of Th17 cytokines. Thus, RORγt is an ideal and attractive target to manipulate in Th17 cell mediated inflammatory diseases^10^. To this end, several small molecules including digoxin and its derivatives^11,12^, diphenylpropanamide compounds^13^, benzenesulfoamide^14–17^, ursolic acid^18^ and compounds containing benzhydryl amide moiety^19–21^ have shown to inhibit RORγt transcriptional activity along with decreased IL-17A and IL-17F production and exhibit significant beneficial effects in animal models of Th17 mediated inflammatory diseases. Recently, small molecule RORγt inverse agonists have also been discovered and are shown to deviate RORγt away from inflammatory signaling^22–24^. However, these compounds are not RORγt specific, but rather affecting ROR family of transcription factors and whereby they will produce undesired adverse effects. In contrast, RNAi based blockade of RORγt inhibition provides more specific suppression of Th17 cells^25^ and is more desirable to clinical development for precision therapy. But critical challenge associated with therapy using RNAi technology is specifically and effectively transfer of small interference RNA (siRNA) or short hairpin RNA (shRNA) across the Th17 cellular membrane. It has been demonstrated that aptamer based delivery system can effectively delivery siRNA into CD4^+^ T cells to inhibit replication of human immunodeficiency virus^26,27^, moreover, we have shown that CD4 specific RNA aptamer can serve as a delivery vehicle to deliver RORγt into primary human CD4^+^ T cells and suppress IL-17 production^25^. The drawback of using CD4 aptamers as delivery vehicle is that they bind CD4^+^ resting T cells and probably poses an unwanted effect. CD4 expressed by monocytes will compete with CD4^+^ T cells in up-taking CD4 aptamers. To overcome these issues, we produced CD30 aptamer-RORγt shRNA (CD30-AshR-RORγt) to suppress RORγt expression. CD30 is expressed by activated T cells including Th17 cells^28–30^ and hence is more specifically target on activated T cells and may represent a desirable aptamer-shRNA chimera for development for Th17 targeted therapy.

## Materials and Methods

### CD30-AshR-RORγt chimera synthesis by *in vitro* transcription

CD30-AshR-RORγt chimera was synthesized as previously described^25^. In brief, commercial DNA oligos (Table 1) were purchased (Integrated DNA Technologies). cDNA templates holding T7 promoter used for synthesis of RNA chimeras were generated by PCR with Pfu DNA polymerase (Thermo Fisher Scientific) and purified with QIAquick Gel purification kit (Qiagen). The sequences of cDNA were verified by Sanger sequencing. Then, the RNA CD30-AshR-RORγt chimera was transcribed *in vitro* by T7 polymerase with DuraScribe kit (Illumina). 2′-FdCTP and F’-dUTP were incorporated to strengthen its resistance to RNases. Cy3-CTP (GE) was incorporated (Cy3-CTP/2′-FdCTP ratio = 1/9) for imaging and resolved on 10% dPAGE Gel for Cy3 scanning and then ethidium bromide staining prior to purification with P-30 micro Bio-Spin chromatography columns (Bio-Rad) and phenol extraction and sodium acetate/ethanol precipitation. The sequences of chimeras of CD30-AshR-RORγt and scrambled shRNA are shown in Table 2. In order to verify if the CD30-AshR-RORγt transcribed *in vitro* is a substrate for endoribonuclearase Dicer that catalyzes cleavage of longer endogenous RNA precursors into short RNA as an intracellular step of RNAi pathway, chimera cleavage was assayed *in vitro* with recombinant human Dicer Kit (Genlantis) in accordance to the guideline. Prostate specific membrane antigen (PSMA)-AshR-RORγt chimera was synthesized as previously described^26,31^.

**Table 1:** DNA oligoes for CD30-AshR-RORγt chimera and CD30-AshR-negative control chimera.

DNA oligo	Sequence
5′-T7-CD30-AshR-RORγt	5′-TAATACGACTCACTATAGGGATTCGTATGGGTGGGATCGGGAA GGGCTACGAACACCGCAATCTCTCTTA-3′
3′-CD30-AshR-RORγt	5′-AACAATCTCTCTTATCCTTGAAAGCACATCAAGGATAAGAGAG ATTGCGGTGTTCGTAG-3′
5′-T7-CD30-AshR-negative	5′-TAATACGACTCACTATAGGGATTCGTATGGGTGGGATCGGGAA GGGCTACGAACACCGGTTCCTCCTAAC-3′
3′-CD30-AshR-negative	5′-AAGTTCCTCCTAACCTATTTAAAGCACATAAATAGGTTAGGAG GAACCGGTGTTCGTAG-3′

**Table 2:** CD30-AshR-RORγt chimera and CD30-AshR-negative control chimera.

Chimera	RNA sequence
CD30-AshR-RORγt	5′-GGGAUUCGUAUGGGUGGGAUCGGGAAGGGCUACGAACACCG CAAUCUCUCUUAUCCUUGAUGUGCUUUCAAGGAUAAGAGAGAU UGUU-3′
CD30-AshR-negative control	5′-GGGAUUCGUAUGGGUGGGAUCGGGAAGGGCUACGAACACCG CAAUCUCUCUUAUCCUUGAUGUGCUUUCAAGGAUAAGAGAGAU UGUU-3′

### T cell lines and T-enriched PBMCs

Karpas 299 and Jurkat cell lines were obtained from Dr. Zu (Houston Methodist Hospital Research Institute) and maintained in RPMI1640 containing 10% FBA. PE-anti-human CD30 (BioLegend) was used to assay expression of CD30 in Karpas 299 and Jurkat cells. For Binding assay, Karpas 299 and Jurkat cells were incubated with 10, 30, 300 and 1000 nM of Cy3-labeled CD30-AshR-RORγt chimera for 1 hour. For up-taking assay, Karpas 299 and Jurkat cells were incubated with Cy3-labeled chimera overnight. To silence RORγt, Karpas 299 cells were incubated with chimeras for 72 hours. PBMCs from healthy donors (n = 6) and RA patients (n = 6) prepared by using Ficoll (GE) density centrifugation and purchased from Astarte Biologics. PBMCs were maintained in RPMI 1640 containing 10% human AB serum. T-enriched PBMCs were prepared and activated with biotinylated antibodies against CD3 and CD28, conjugated to anti-biotin MASC beads (Miltenyi Biotech Inc) and 100 ng/ml lipopolysaccharide (LPS), as described previously^25^. For intracellular staining of RORγt and IL-17A and IL-17F, PBMCs were harvested prior to incubating with PMA (50 ng/ml), inomycin (50 ng/ml) and BD GolgiPlug^TM^ protein transporter inhibitor (eBiosciences).

### Flow cytometry and fluorescent microscopy

CD30 surface staining on Karpas 299 cells and Jurkat cells was analyzed with flow cytometry (Fortessa, BD Sciences) after incubation of PE-anti-human CD30 antibody (eBioscience). CD4 in PBMCs was staining with APC/FITC-anti-human CD4 and analyzed with fluorescent microscopy and flow cytometry. Binding of the Cy3-labeled chimeras on Karpas 299 and Jurkat cells was determined by flow cytometry. Uptake of Cy3-labeled chimeras by Karpas 299, Jurkat cells and PBMCs was analyzed with fluorescent microscopy. Intracellular cellular staining for RORγt, IL-17A and IL-17F in Karpas 299 cells and PBMCs was determined with flow cytometric analysis after cells were fixed and permeable with fixation/permeabilization buffer (eBioscience) and then incubated with PE-anti-mouse/human RORγt and PE-anti-human IL-17A and PE-Cy7-anti-IL-17F (eBioscience).

### Real-time PCR

Real-time PCR was carried out as previously described^25^. Total RNA was prepared with RNeasy mini Kit (Qiagen). The probes and primers for RORγt and GUSB were purchased from Thermo Fisher Scientific. mRNA levels of RORγt were normalized by GUSB.

### Statistics

Data presented here were mean ± SD. Data of real-time PCR, and flow cytometry were analyzed by one-way ANOVA followed by Dunnett comparison test. If *P* value was less than 0.05, the differences were considered to be significant.

## Results

### Synthesis of CD30-AshR-RORγt chimera

**Figure 1:**
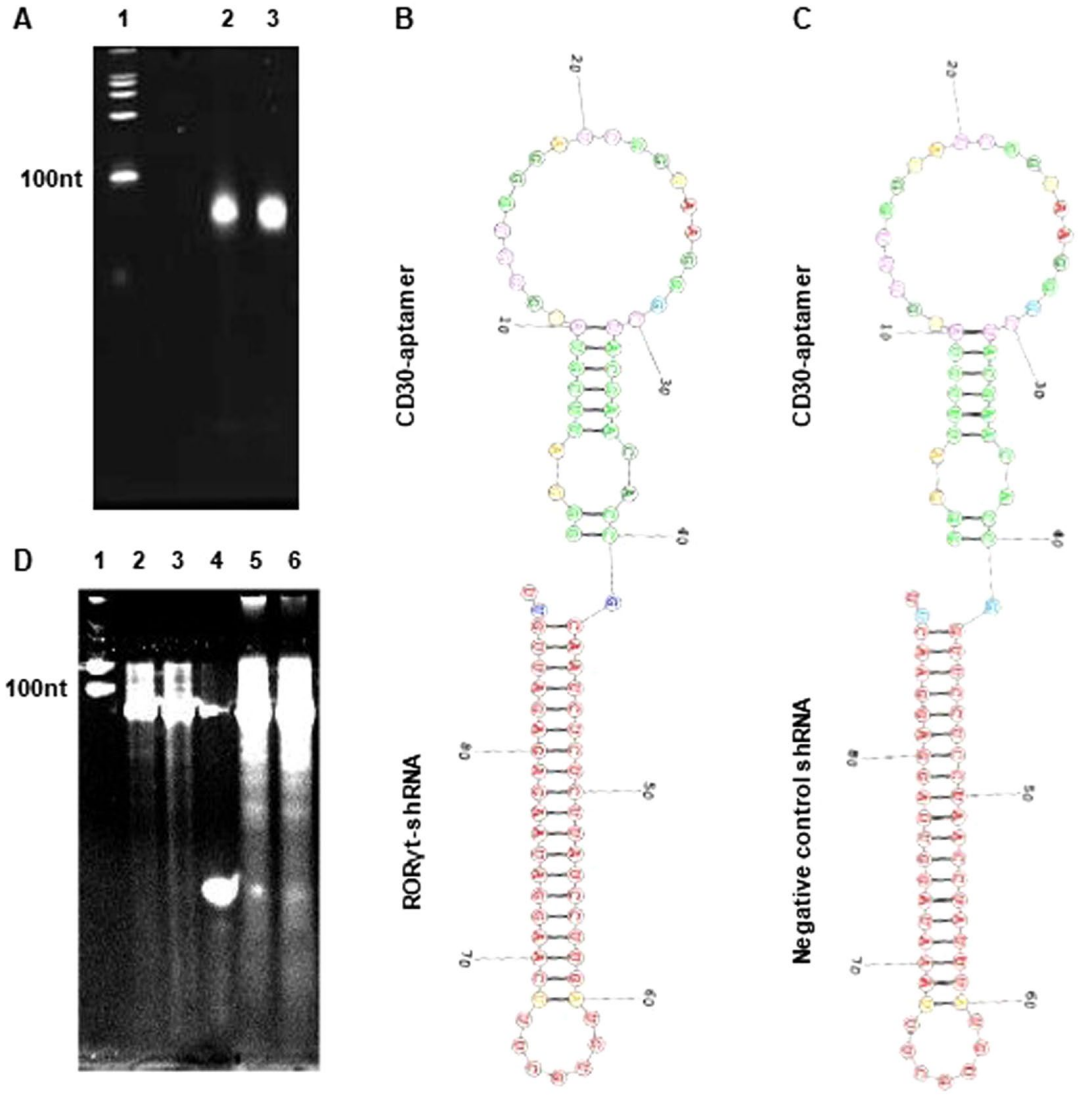
CD30-AshR-RORγt chimera. (**A**) Chimera *in vitro* transcribed by T7 RNA polymerase was analyzed by denatured PAGE and ethidium staining. Lane 1, ssRNA standard ladder; Lane 2, CD30-AshR-RORγt chimera; Lane 3, CD30-AshR-negative control chimera. (**B**,**C**) Predicted second structures of CD30-AshR-RORγt chimeras and CD30-AshR-negative control chimera. The region of the CD30 aptamer responsible for binding to CD30 is outlined. The shRNA portion of the chimera consists of targeted RORγt shRNA with 2 overhang nucleotides at its 3′ end and 7 nucleotide loop. (**D**) Cleavage analysis of synthesized chimeras by Dicers. Lane 1, ssRNA standard ladder; Lane 2, intact CD30-AshR-RORγt chimera; Lane 3, intact CD30-AshR-negetive control chimera; Lane 4, antisense siRNA; Lane 5, CD30-AshR-RORγt chimera digested with Dicers; Lane 6, CD30-AshR-negetive control chimera digested with Dicers.

CD30-AshR-RORγt chimera consists of CD30 aptamer and shRNA against RORγt (Fig. 1). CD30-AshR-negative control chimera consists of CD30 aptamer and shRNA for a scrambled sequence. CD30-AshR-RORγt and CD30-AshR-negative control chimeras were generated with T7 transcription. Both were 88 nucleotide in length (Fig. 1). The predicted secondary structures for CD30-AshR-RORγt and CD30-AshR-negative control chimeras are produced with the RNA structure software of David H. Mathew’s laboratory (University of Rochester) (Fig. 1B,C). Cleavage analysis of both synthesized CD30-AshR-RORγt chimeras and CD30-AshR-negative control chimeras by Dicers is shown (Fig. 1D).

### Specific binding and internalization of Cy3-CD30-AshR-RORγt chimera by CD30-expressing Karpas 299 cells and activated CD4^+^ T cells

**Figure 2:**
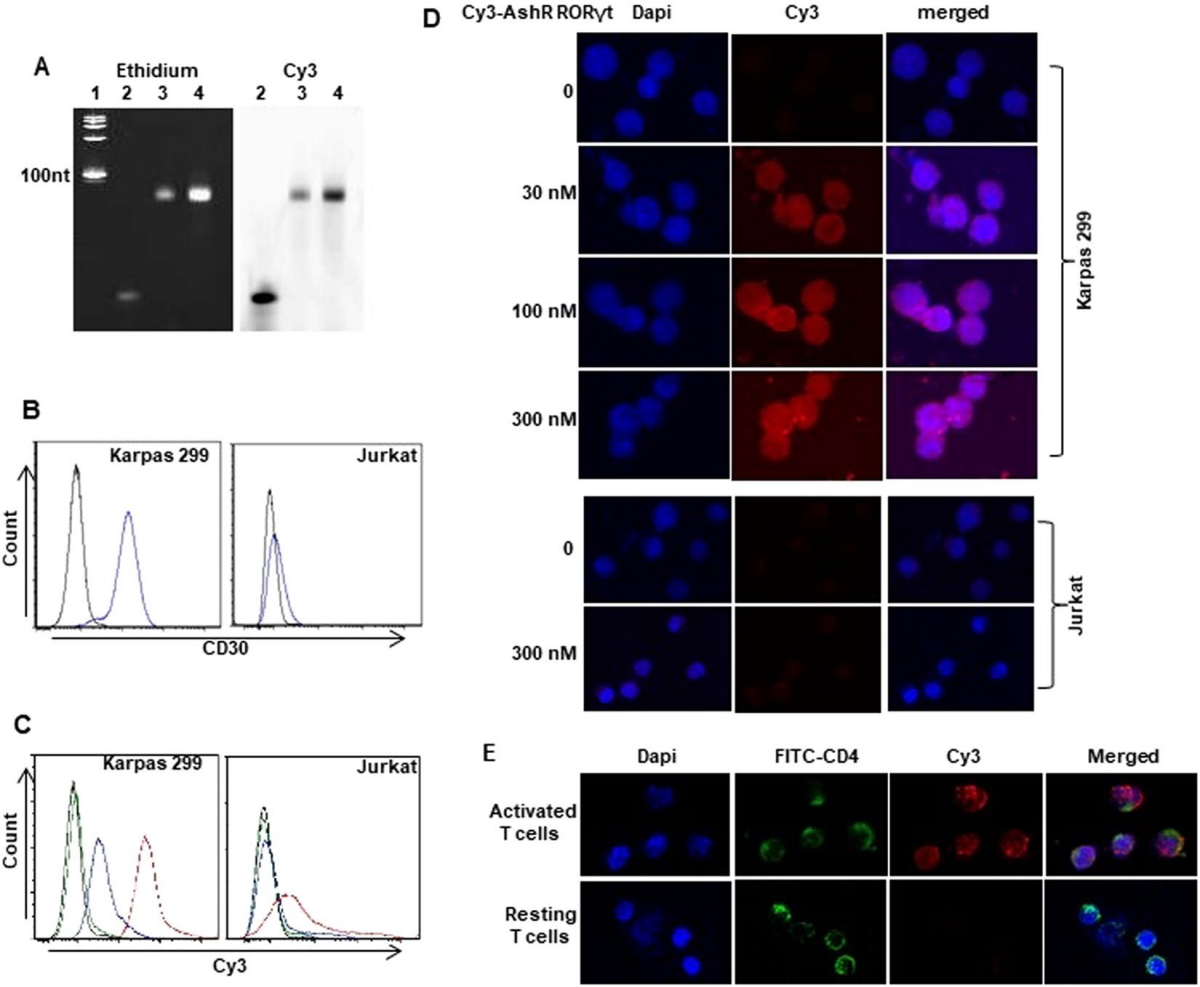
CD30-AshR-RORγt chimera selectively bound and entered human CD30^+^ T cells. (**A**) CD30-AshR-RORγt chimera was labeled by incorporating Cy3-CTP during *in vitro* transcription. Cy3 scanning (right panel) displayed strong Cy3 signaling band that was at appropriate size of transcript shown in ethidium imaging (left panel). Lane 1, ssRNA standard Ladder; Lane 2 and lane 3, 200 and 400 ng of Cy3 -CD30-AshR-RORγt chimeras. (**B**) CD30 is highly expressed in Karpas 299 cells while CD30 is weakly expressed in Jurkat cells. (**C**). Concentration-dependent binding of Cy3-labeled CD30-AshR-RORγt chimeras by human CD30^+^ T cells. Cy3-labeled CD30-AshR-RORγt chimeras binding in Karpas 299 cells (Left panel) and a weak binding of 300 nM Cy3-labeled CD30-AshR-RORγt chimera was observed in Jurkat cells (Right panel). Compared to Karpas 299 cells, there was significantly less binding of Cy3-CD30-AshR-RORγt chimeras by human T cell line Jurkat cells. Black line, control; Green, 3 nM; Blue, 30 nM and Red 300 nM. (**D**) Confocal microscopic images show that Cy3-CD30-AshR-RORγt chimera was up-taken by Karpas 299 cells after overnight incubation in a concentration dependent manner. There is no significant internalization of Cy3-CD30-AshR-RORγt chimera in Jurkat cells. (**E**) Confocal images showed that Cy3-CD30-AshR-RORγt chimera was up-taken by activated human CD4^+^ T cells after overnight incubation in a concentration dependent manner. There is no internalization of Cy3-CD30-AshR-RORγt chimera in resting human CD4^+^ T cells (Representative of 2–5 experiments).

For imaging analysis, Cy3-dCTP was incorporated during synthesis of CD30-AshR-RORγt chimera. Successful incorporation of Cy3-dCTP into CD30-AshR-RORγt chimera was identified by fluorescent scanning (Fig. 2A). Surface CD30 was verified in Karpas 299 cells, but only a weak expression of CD30 was observed in Jurkat cells (Fig. 2B). Consistent with highly expressed CD30, Karpas 299 cells displayed a significantly concentration-dependent binding of Cy3-CD30-AshR-RORγt chimera, while Jurkat cells expressing a little CD30 only exhibited a very weak binding at high concentration of Cy3-labeled chimera (Fig. 2C), suggesting that CD30-AshR-RORγt chimera preserves specific binding to CD30 as original CD30 aptamer though short hairpin RNAs are linked. Furthermore, a concentration-dependent entry of Cy3-CD30-AshR-RORγt chimera into Karpas 299 cells was verified after overnight incubation, but not in Jurkat cells (Fig. 2D). Also, Cy3-CD30-AshR-RORγt chimera was internalized into activated human CD4^+^ T cells, but not in resting CD4^+^ human T cells, as assessed by confocal microscopy (Fig. 2E).

### Silence of RORγt in Karpas 299 cells and activated human CD4^+^ T cells by CD30-AshR-RORγt chimera

**Figure 3:**
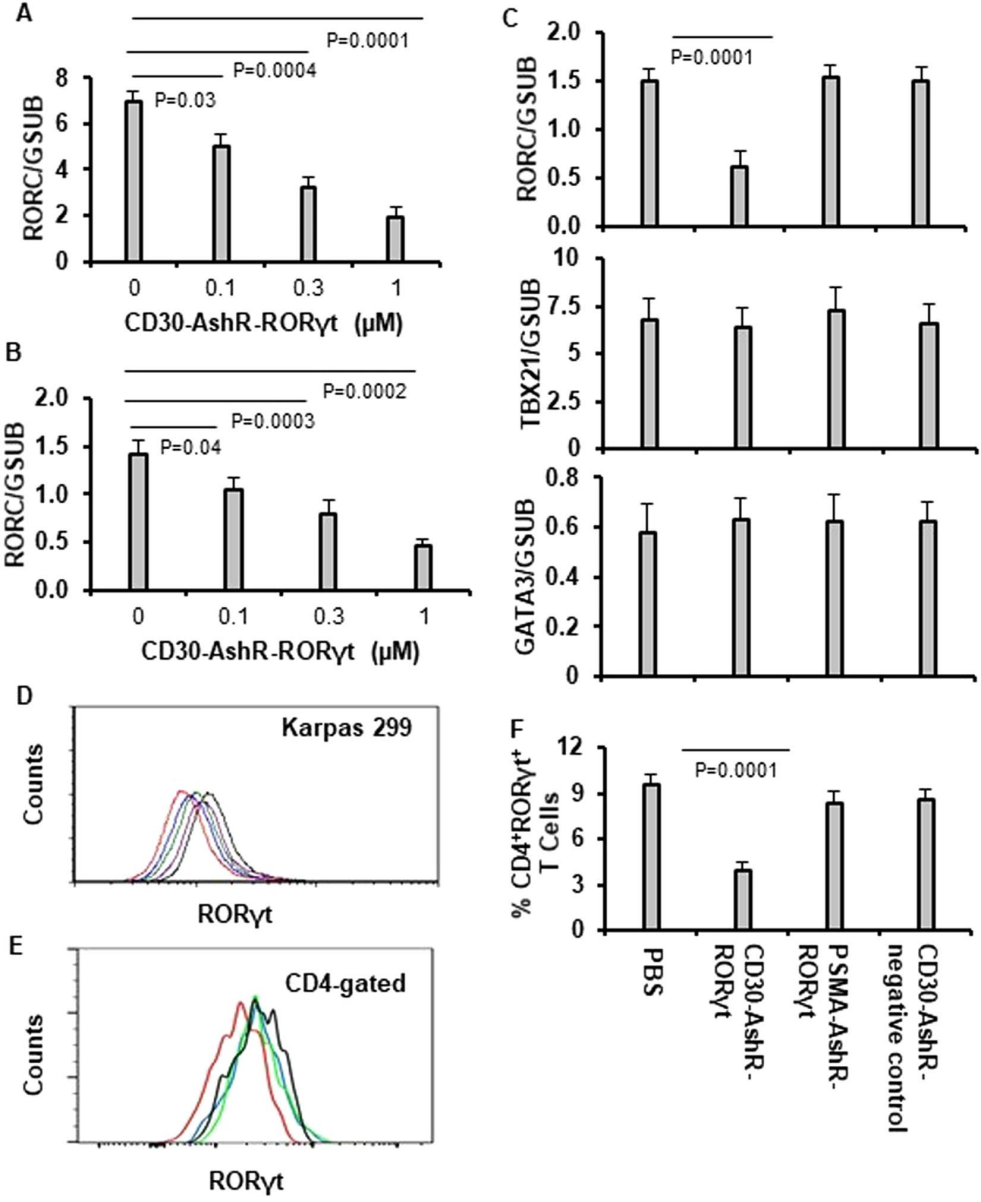
Specific silencing of RORγt in Karpas cells and activated human CD4^+^ T cells by CD30-AshR-RORγt chimeras. Karpas 299 cells were treated as described in the methods. (**A** though **C**) Quantitative real-time PCR assay for the gene expression of RORγt, TBX21 and GATA3. CD30-AshR-RORγt chimera significantly diminished RORγt gene expression in a concentration dependent manner in Karpas 299 cells (**A**) and activated human T enriched PBMCs (**B**). CD30-AshR-negative control chimera and PSMA-AshR-RORγt chimera had no impact on RORγt gene expression in activated T enriched PBMCs. These chimeras did not affect the TBX21 and GATA3 gene expression in activated human T enriched PBMCs (**C**). (Data are presented as mean ± SD of 4 experiments). (**D**,**E**) RORγt protein levels were assessed by flow cytometry. CD30-AshR-RORγt chimera reduced RORγt protein levels in a concentration-dependent fashion in Karpas 299 cells (**D**) and in PBMCs (**E**). Black line, PBS, Purple line 0.03 µM, Green line, 0.1 µM, Blue line 0.3 µM, Red line 1 µM (Representative histogram of of 4 experiments). (**F**) CD30-AshR-RORγt chimera reduced the percentages of CD4^+^RORγt^+^ cells in activated human T enriched PBMCs, however PSMA-AshR-RORγt and CD30-AshR-negative control chimeras had no impact(mean ± SD of 4 experiments).

Real-time PCR showed that CD30-AshR-RORγt chimera resulted in concentration-dependent decrease of RORγt mRNA levels in Karpas 299 cells (Fig. 3A). Consistent with this, CD30-AshR-RORγt chimera significantly reduced intracellular RORγt in Karpas 299 cells in a concentration-dependent manner (Fig. 3D), as analyzed with flow cytometry. Similarly, CD30-AshR-RORγt chimera caused a significant and concentration-dependent reduction of RORγt mRNA in activated human PBMCs (Fig. 3B). PSMA-AshR-RORγt chimera and CD30-AshR-RORγt chimera had no effect on RORγt mRNA in activated human PBMCs (Fig. 3C left panel). None of the three chimeras affected mRNA levels of TBX21 and GATA3 in activated human PBMCs (Fig. 3C middle and right panels), indicating the specificity of RORγt shRNA. Also, the percentage of CD4^+^RORγt^+^ T cells in activated human PBMCs were significantly impaired by CD30-AshR-RORγt chimera, but CD30-AshR-negative control chimera and PSMA-AshR-RORγt chimera had no effect (Fig. E, histogram representative of four experiments; Fig. 3F, mean ± SD of four experiments), suggesting that CD30-AshR-RORγt chimera exerts a significant impact in targeting and silencing RORγt of activated human CD4^+^ T cells.

### Suppression of IL-17A and IL-17F synthesis in human CD4^+^ T cells from healthy donors and RA patients by CD30-AshR-RORγt chimera

**Figure 4:**
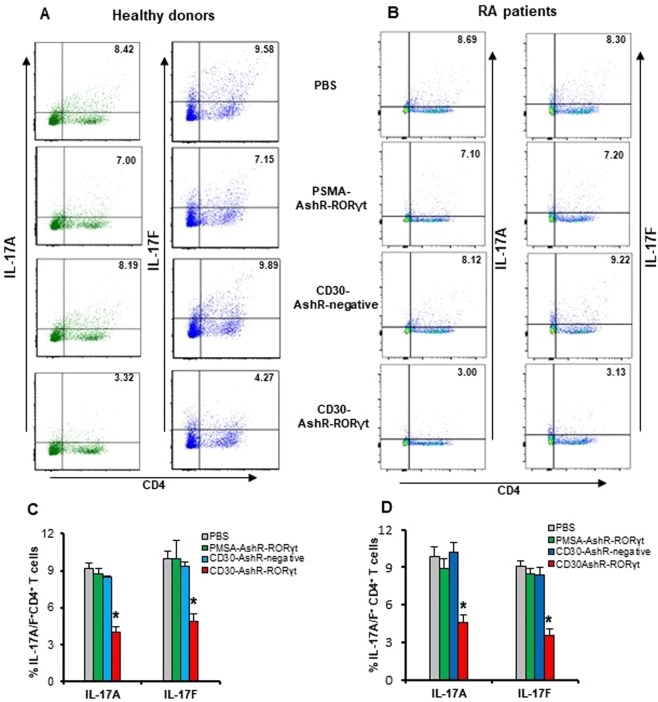
Decrease of IL-17A and IL-17F synthesis in activated human CD4^+^ T cells by CD30-AshR-RORγt chimera. T-enriched PBMCs were stimulated with anti-CD3/CD28 and LPS and treated as described in the Materials and Methods. (**A**,**C**) CD30-AshR-RORγt chimera significantly diminished percentage of CD4^+^IL-17A^+^ cells and CD4^+^IL-17F^+^ cells in stimulated T enriched PBMCS from healthy donors, but CD30-AshR-negative control or PSMA-AshR-RORγt chimera lacked the effect. Data are mean ± SD of 4 experiments. (**B**,**D**) Similarly, CD30-AshR-RORγt chimera also significantly impaired percentage of CD4^+^IL-17A^+^ cells and CD4^+^IL-17F^+^ cells in stimulated T enriched PBMCS from RA patients, but CD30-AshR-negative control or PSMA-AshR-RORγt chimera had no impacts. *p < 0.05 versus PBS, Data are mean ± SD of 4 experiments.

RORγt is a master transcription factor that controls Th17 subtype differentiation and synthesis of Th17 cytokines. Thus, it is expected that silencing RORγt by CD30-AshR-RORγt chimera will inhibit synthesis of Il-17A and IL-17F as well. As shown in Fig. 4A,B, CD30-AshR-RORγt significantly decreased intracellular staining of IL-17A and IL-17F by 60% in activated human CD4^+^ T cells from healthy donors, but CD30-AshR-negative control chimera and PSMA-AshR-RORγt chimera had no effects. It was similarly observed in activated human CD4^+^ T cells from RA patients that CD30-AshR-RORγt significantly suppressed IL-17A and IL-17F as demonstrated by decreased intracellular staining of these cytokines, while CD30-AshR-negative control chimera and PSMA-AshR-RORγt chimera had no effects on IL-17A or IL-17F synthesis (Fig. 4C,D). In order to rule out the possibility that reduction of IL-17A and IL-17F was due to cell death, the total number of cells in the culture was numerated and the number of CD4^+^ cells was derived from percentage of CD4^+^ cells in flow cytometry analysis. There were no significant difference in total CD4^+^ T cells observed between treatment groups. These results suggest that reduction of IL-17A and IL-17F producing cells was the result of suppression of RORγt expression rather than cell death.

## Discussion

Pathogenic effector Th17 cells are highly desirable target for treating Th17 cell mediated diseases. However, the precise targeting is challenging. Current available therapeutics are targeting individual Th17 cytokines, but not the pathogenic Th17 cells. The ideal agent will need to specifically and directly act on Th17 cells to down regulate Th17 cytokines and spare these cytokines produced by other immune cells^32^. Recently, several small molecules have been shown to be able to suppress or deviate the signaling of the Th17 master transcription factor, RORγt, but these small molecules are not Th17 cell specific nor RORγt specific^10^.

RNAi based technology provides gene specific target but suffers from insufficient therapeutic level of delivery to the target cell types. Aptamers have been explored to serve as delivery vehicles to transfer siRNA or shRNA into target cells. A target cell specific aptamer is linked with an siRNA or shRNA to form a molecule complex which is then internalized by the target cell. Several methods are reported to link an aptamer with an siRNA or shRNA for the purpose of delivery^33,34^. McNamra *et al*.^31^ pioneered in making an aptamer-siRNA chimeric RNA, where an RNA aptamer specific for PSMA was directly connected with siRNA targeting survival genes for cancer therapy. This approach was adopted by Wheeler *et al*.^26^ and made a CD4 aptamer-CCR5 siRNA and/or CD4 aptamer-HIV siRNA for inhibition of HIV replication in an *in vivo* model. We have made a CD4 aptamer-shRNA for RORγt to inhibit Th17 cells^25^. The aptamer-shRNA chimera has significant technical advantage versus aptamer-siRNA. In aptamer-siRNA chimera, the single chained aptamer-anti-sense of the siRNA is transcribed and then the sense strand of the siRNA is annealed. This can be technically challenging since the annealing rate varies. Whereas, aptamer-shRNA is produced by one stage transcription and the production is readily high yield. The major barrier for *in vivo* use of CD4-AshR-RORγt was the uptake by monocytes which expressing low levels of CD4.

Since there is no cell surface molecule that has been identified to be specific for pathogenic Th17 cells, we took an alternative approach by using CD30 as a portal to deliver shRNA to RORγt. CD30 RNA aptamer that was developed by Mori *et al*. recognizes a common structure conserved in the TNF receptor family proteins, rather than the ligand-binding site and has a very high affinity of binding CD30 with dissociation constant 0.11 nM^35^. CD30 aptamer-based probes were synthesized and used for imaging of tumors expressing biomarker CD30, further confirming the binding specificity of CD30 RNA aptamers with high affinity^36^. Here we show that addition of RORγt shRNA did not affect the CD30 aptamer forming secondary structure. Indeed, the CD30-AshR-RORγt chimera shows specific binding to CD30 expressing tumor cells and activated primary human T cells expressing CD30 and successfully delivered shRNA to suppress RORγt. The silencing effects of CD30-AshR-RORγt chimera on *Rorc* gene expression are consistent with specific siRNA transfected by lipid transfection agents^37^. Importantly, CD30-AshR-RORγt chimera is specific for *Rorc* gene since *Tbox 21* and *Gata3* genes in CD4^+^ T cells were not affected, suggesting that CD30-AshR-RORγt chimera does not have off-target effects.

In summary, the data of this study revealed that CD30 can serve as a delivery vehicle for shRNA that targets specific gene, RORγt in CD30 expressing activated human T cells. The internalized RORγt shRNA via CD30 aptamer can be cleavage by Dicers and then specifically silence RORγt gene expression and led to marked decrease of Th17 cell differentiation and IL-17A and IL-17F synthesis. Therefore, CD30-AshR-RORγt chimera may potentially be developed into a therapeutic approach to treatment of autoimmune inflammatory diseases such as RA.
